# Book Report: Health Economics and Pharmacoeconomics for the Students of Medical Sciences; ISBN 978-86-7760-082-2

**DOI:** 10.3389/fpubh.2016.00242

**Published:** 2016-11-07

**Authors:** Dejana M. Savić

**Affiliations:** ^1^Faculty of Medical Sciences, University of Kragujevac, Kragujevac, Serbia

**Keywords:** health economics, pharmacoeconomics, textbook, students, medicine, Serbia, western Balkans

I have been honored by privilege of presenting a recent university textbook written in Serbian language entitled: “Health Economics and Pharmacoeconomics for the Students of Medical Sciences,” Edited by Prof. Mihajlo (Michael) Jakovljevic MD, PhD (1). This book was authored by 38 leading experts in their respective fields, based in South-Eastern Europe. Participation of governmental, academic, and industrial sector among the authors of chapters helped to provide overview of major health economics issues from various aspects. This book is an ambitious pioneer project in creating university textbook for postgraduate students in this field in native Balkan languages. Hereby, I will provide a short description of major topics covered by the Book content.

This textbook has at first been intended for use by undergraduate students of medicine, pharmacy, and dentistry to cover an existing knowledge gap in applied health sciences. However, it turned out to be of an immense practical use also for postgraduate master and PhD students of interdisciplinary health sciences due to comprehensive interdisciplinary reach. Due to its lingual limitations, this textbook is of use only in areas of mostly mutually understandable Western Balkan languages: Serbian, Slovenian, Croatian, Bosnian, and Macedonian. This textbook should be considered as highly recommendable for studying and research by students of most private-owned and state-owned universities and other higher education establishments throughout former Yugoslavia region.

The Book consists of 68 chapters (631 pages) organized in five consecutive sections. General Part presents introduction to the concepts and definition of health-care economics. Its historical development and terminology of the discipline is presented in further exposé. The second, Special Part exposes the issues of cost-of-illness studies as seen by various leading clinical medicine experts familiar with the respective fields. Several chapters consider health-care budget impact of some of the most costly medical interventions. Consecutive Pharmacoeconomics Part is presented in the form of detailed and elaborated discussion about different methodological approaches ranging from cost-effectiveness assessments to resource allocation analysis. Conclusive Remarks describe and elaborate the emerging markets and provide quite realistic picture of the expected developments among the Global and Eastern European/SEE health-care markets. Fifth part is formed as the Book of Terms, Index, and comprehensive English/Serbian vocabulary of essential standardized and broadly used international terms in disciplines of health economics and health policy. This segment of the project should be of immense value for regional health economists and policy makers understanding these topics and dealing with them in every day routine. Consensus of vast variety of professionals of the respective fields is expected to diminish presumed lingual, semantic, and lexical difficulties that arise from diverse variations in translation of English-born notions to the Western Balkan languages.

It would have been most certainly of great benefit if authors had considered discussing paperwork issues that are very common in countries in transition even in routine procedures, let alone in new projects, no matter their routine implementation in developed countries.

As to be concluded on presented material, it is clear that this challenging endeavor was performed in line with high academic standards and principles of good editorial practice. The textbook entitled: “Health Economics and Pharmacoeconomics for the Students of Medical Sciences” will certainly be of use for generations of graduate and PhD students as well as the teaching staff and research communities of the Western Balkans region. Combined efforts of broad spectrum of professionals from academia, industry, and national authorities have greatly contributed to the Book’s quality and its comprehensive reach. Energized developments in health-care economics are induced by the emerging Eastern European markets undergoing end stages of transitional health reforms (2). Such changes in health-care financing, provision, and management patterns will inevitably affect academic aspects of health-care economics. It is also of a great importance to mention an exceptional contribution of this book as a textbook of health-care field of expertise as such in general.

This landscape implicates requirement for new knowledge to cope with the upcoming challenges (3–6). This pioneering achievement in academic health economics of SEE region might serve as a prototype for future Endeavor of a kind.

## Author Contributions

DS: conception and design, writing the article, critical revision of the article, final approval of the article, and overall responsibility.

## Conflict of Interest Statement

The author declares that the research was conducted in the absence of any commercial or financial relationships that could be construed as a potential conflict of interest.
